# Early language intervention and IQ of children with non-syndromic orofacial clefts

**DOI:** 10.1192/j.eurpsy.2024.937

**Published:** 2024-08-27

**Authors:** K. A. Sándor-Bajusz, E. Molnár, T. Dergez, K. Hadzsiev, A. Vástyán, G. Csábi

**Affiliations:** ^1^Pediatrics, Division of Child and Adolescent Psychiatry; ^2^ Institute of Bioanalysis; ^3^Department of Medical Genetics; ^4^Pediatrics, Division of Pediatric Surgery, University of Pécs, Medical School and Clinical Center, Pécs, Hungary

## Abstract

**Introduction:**

Children with non-syndromic orofacial clefts are at higher risk for developmental difficulties. Speech and language as commonly affected developmental domains in these children.

**Objectives:**

The aim of the current study was to explore the effects of early interventions for speech and language on later cognitive outcomes in this patient population.

**Methods:**

A combined retrospective/prospective-comparative study was carried out at the Department of Pediatrics of the University of Pécs in Hungary. The participants were children between 6 and 16 years of age. The study consisted of a self-designed demographic questionnaire and an IQ test (WISC-IV).

**Results:**

A total of 41 children with non-syndromic orofacial clefts and 44 age-matched controls participated in the study. Children of the cleft group were examined by pedagogical professional services and required special education plans significantly more often than controls (p<0.001 and p=0.02, respectively). Participants of the cleft group who received early speech and language therapy score higher on the Verbal Comprehension Index (p=.005). Full-Scale IQ score was also higher for cleft participants who received therapy, however not significant but borderline (p=0.08).

**Conclusions:**

Early language and speech interventions for children with non-syndromic orofacial clefts may have a positive effect on verbal skills and overall cognitive development. Future longitudinal studies examining baseline cognitive functioning of infants are needed to provide more conclusive evidence on the effects of interventional programs on speech and language development in cleft patients.

**Disclosure of Interest:**

None Declared

